# Heat shock protein expression in canine malignant mammary tumours

**DOI:** 10.1186/1471-2407-6-171

**Published:** 2006-06-27

**Authors:** Mariarita Romanucci, Alessia Marinelli, Giuseppe Sarli, Leonardo Della Salda

**Affiliations:** 1 Department of Comparative Biomedical Sciences, Faculty of Veterinary Medicine, University of Teramo, Teramo, Italy; 2 Department of Veterinary Public Health and Animal Pathology, Faculty of Veterinary Medicine, University of Bologna, Bologna, Italy

## Abstract

**Background:**

Abnormal levels of Heat Shock Proteins (HSPs) have been observed in many human neoplasms including breast cancer and it has been demonstrated that they have both prognostic and therapeutic implications. In this study, we evaluated immunohistochemical expression of HSPs in normal and neoplastic canine mammary glands and confronted these results with overall survival (OS), in order to understand the role of HSPs in carcinogenesis and to establish their potential prognostic and/or therapeutic value.

**Methods:**

Immunohistochemical expression of Hsp27, Hsp72, Hsp73 and Hsp90 was evaluated in 3 normal canine mammary glands and 30 malignant mammary tumours (10 *in situ *carcinomas, 10 invasive carcinomas limited to local structures without identifiable invasion of blood or lymphatic vessels, 10 carcinomas with invasion of blood or lymphatic vessels and/or metastases to regional lymph nodes). A semi-quantitative method was used for the analysis of the results.

**Results:**

Widespread constitutive expression of Hsp73 and Hsp90 was detected in normal tissue, Hsp72 appeared to be focally distributed and Hsp27 showed a negative to rare weak immunostaining. In mammary tumours, a significant increase in Hsp27 (P < 0.01), Hsp72 (P < 0.05) and Hsp90 (P < 0.01) expression was observed as well as a significant reduction in Hsp73 (P < 0.01) immunoreactivity compared to normal mammary gland tissue. Hsp27 demonstrated a strong positivity in infiltrating tumour cells and metaplastic squamous elements of invasive groups. High Hsp27 expression also appeared to be significantly correlated to a shorter OS (P = 0.00087). Intense immunolabelling of Hsp72 and Hsp73 was frequently detected in infiltrative or inflammatory tumour areas. Hsp90 expression was high in all tumours and, like Hsp73, it also showed an intense positivity in lymphatic emboli.

**Conclusion:**

These results suggest that Hsp27, Hsp72 and Hsp90 are involved in canine mammary gland carcinogenesis. In addition, Hsp27 appears to be implicated in tumour invasiveness and its high immunodetection in invasive tumours is indicative of a poorer clinical outcome.

## Background

Heat Shock Proteins (HSPs) or Stress Proteins are one of the most evolutionarily conserved classes of molecule and play a fundamental role in the maintenance of cellular homeostasis. Under physiological conditions, they act as "molecular chaperones", by assisting protein folding, transport and degradation; during stress, on the other hand, they prevent aggregation and promote refolding of damaged proteins. HSPs are classified into several families, named according to their approximate molecular weight [1]. The 27-kDa Heat Shock Protein (Hsp27) is an actin-associated protein, predominantly expressed in skin and cells of normal estrogen target organs [2], whereas the HSP70 family includes constitutive (Hsc70 or Hsp73) and inducible (Hsp70 or Hsp72) members, localized within both the cytoplasm and the nucleus of all tissue cells [1,3]. Hsp90 is one of the most abundant proteins in mammalian cells [4] and is made up of two isoforms, Hsp90α (inducible/major form) and Hsp90β (constitutive/minor form) [5]. Tumour cells appear to be dependent on increased levels of these proteins, since elevated expression of different HSPs has been observed in several human neoplastic conditions [6]. Members of Hsp27, Hsp70 and Hsp90 families are thought to play a role in breast cancer [7]: the possible relationship between their abnormal expression and the prognosis of the disease [8-17] or the responsiveness to a specific therapy [10,18] has been studied extensively. It has also been suggested that HSPs are directly implicated in the drug resistance of breast cancer cells [19,20]. Nevertheless, HSPs appear to be promising therapeutic targets for several kinds of tumours, including breast cancer [21-26].

In veterinary medicine, mammary tumours constitute the most common malignant neoplasms in the bitch [27]; furthermore, they show wide pathological and clinical heterogeneity similarly to the disease in humans. Investigations aimed at discovering clinical and pathologic parameters with prognostic and/or therapeutic significance in these neoplasms are considered an important field of study in this species [28], also in light of the potential usefulness of the animal model for the study of human breast cancer.

The aims of this study were to evaluate the immunohistochemical expression of different HSPs in canine malignant mammary tumours and to establish whether this expression could be correlated to the histological stage of the neoplasm and overall survival, thus studying the role of these proteins in the carcinogenesis of the mammary gland and establishing their potential prognostic and/or therapeutic implications for this frequent neoplastic condition of the dog.

## Methods

### Histological examination

The study was carried out on necropsy samples from 3 normal canine mammary glands and surgical samples from 30 malignant mammary tumours. Ethical approval for the study was granted by the institutional review board of the Faculty of Veterinary Medicine, University of Teramo (Italy). All tumour cases were supplied by the Department of Veterinary Public Health and Animal Pathology, Faculty of Veterinary Medicine, University of Bologna (Italy). Samples were fixed in 10% neutral buffered formalin and embedded in paraffin wax. Histological diagnosis was established using haematoxylin and eosin-stained slides, according to WHO guidelines [29], while histological stage of infiltrating tumours was determined according to the system proposed by Gilbertson *et al*. [30]. Following these criteria 10 cases revealed *in situ *carcinomas [29], while 20 cases were infiltrating carcinomas further grouped as stage I – invasive carcinoma limited to local structures without identifiable invasion of blood or lymphatic vessels (10 cases) and stage II – invasion of blood or lymphatic vessels and/or metastases to regional lymph nodes (10 cases).

### Immunohistochemistry

Formalin-fixed, paraffin-embedded samples were processed using an immunohistochemical technique with specific anti-Hsp27 (1:600, rabbit polyclonal, StressGen, Victoria, BC Canada), anti-Hsp72 (1:100, C92F3A-5, mouse monoclonal, StressGen), anti-Hsp73 (1:500, 1B5, rat monoclonal, StressGen) and anti-Hsp90 (1:2500, AC88, mouse monoclonal, StressGen) antibodies (Abs).

Deparaffined and rehydrated sections were incubated in 3% hydrogen peroxide in absolute methanol for 45 min in order to inhibit endogenous peroxidase activity, then rinsed in 0.05 M Tris-buffered saline (TBS), pH 7.6, for 5 min. Antigen retrieval was performed by heat-treating sections in citrate buffer at pH 6 in a microwave oven for 5 min (3 cycles). To reduce non-specific binding, slides were incubated in normal goat serum (Biospa, Milan, Italy) for 10 min at room temperature, before overnight incubation with primary Ab in a humified chamber at 4°C. After rinsing with TBS, immune complexes were treated with secondary biotinylated Goat anti-Mouse&Rabbit (ready-to-use, Biospa, Milan, Italy), or Rabbit anti-Rat (1:100, DAKO, Copenhagen, Denmark) Abs and subsequently detected using streptavidin-peroxidase (Biospa, Milan, Italy), both incubated at room temperature for 10 min. Peroxidase activity was detected using 0.1% hydrogen peroxide in 3-3'-diaminobenzidine (DAB) solution (Sigma) applied to the tissue sections for 5 min, which were then counterstained with Papanicolau's haematoxylin for 5 sec before rinsing, dehydrating and mounting with coverslips.

A negative control was performed in all cases by omitting the primary Ab and incubating tissue sections with TBS and/or with an irrelevant antibody directed against an unrelated antigen such as rabbit anti-human von Willebrand factor polyclonal Ab (DAKO, Glostrup, Denmark) or mouse anti-human desmin monoclonal Ab (DAKO, Glostrup, Denmark).

### Clinical follow-up

Survival data concerning the dogs were supplied by the Department of Veterinary Public Health and Animal Pathology, Faculty of Veterinary Medicine, University of Bologna (Italy). Dogs were clinically examined by veterinarians every 3 months after surgical treatment for a minimum of 2 years. Follow-up included a radiological evaluation of the thorax and an ultrasound scan of liver and kidneys. For the animals that died within the two year period, overall survival (OS) was considered the months between surgery and death, whilst for the dogs who survived >2 years, the OS was the number of months from surgery to the last clinical examination. OS data in relation to histological diagnosis and stage are shown in Table 1.

**Table 1:** Histological diagnosis, tumour stage and overall survival of the cases comprised in the present study

	**Histological diagnosis**	**Overall Survival (months)**
n° 1	*in situ *carcinoma	23
n° 2	*in situ *carcinoma	22
n° 3	*in situ *carcinoma	36
n° 4	*in situ *carcinoma	29
n° 5	*in situ *carcinoma	24
n° 6	*in situ *carcinoma	27
n° 7	*in situ *carcinoma	23
n° 8	*in situ *carcinoma	29
n° 9	*in situ *carcinoma	60
n° 10	*in situ *carcinoma	28

**STAGE I**		

n° 1	simple solid carcinoma	24
n° 2	simple tubular carcinoma	27
n° 3	carcinoma in benign tumour	22
n° 4	simple tubulopapillary carcinoma	27
n° 5	carcinoma in benign tumour	22
n° 6	simple solid carcinoma	26
n° 7	simple tubulopapillary carcinoma	33
n° 8	complex carcinoma	24
n° 9	simple tubulopapillary carcinoma	28
n° 10	simple carcinoma	42

**STAGE II**		

n° 1	simple tubulopapillary carcinoma	36
n° 2	simple solid carcinoma	4
n° 3	simple solid carcinoma	1
n° 4	simple solid carcinoma	26
n° 5	simple solid carcinoma	9
n° 6	simple solid carcinoma	24
n° 7	simple solid carcinoma	1
n° 8	carcinoma in benign tumour	12
n° 9	simple tubulopapillary carcinoma	2
n° 10	simple solid carcinoma	26

### Statistical analysis

A semi-quantitative immunohistochemical assessment (absent: no positive cells; low: rare positive cells; intermediate: up to 50% of positive cells; high: over 50% of positive cells) was made comparing normal and malignant mammary gland tissue and, in the latter, comparing groups (*in situ *carcinomas, stage I and II infiltrating carcinomas) by Chi square test. Influence on survival was established using the Log-Rank Test and cases were grouped according to expression: low (absent + low semiquantitative evaluation) or high (intermediate + high semiquantitative evaluation). Analyses were performed using CSS software (Statsoft, Tulsa, OK) statistics, and a conventional 5% level was used to define statistical significance.

## Results

### HSP expression in normal mammary gland

**Figure 1:**
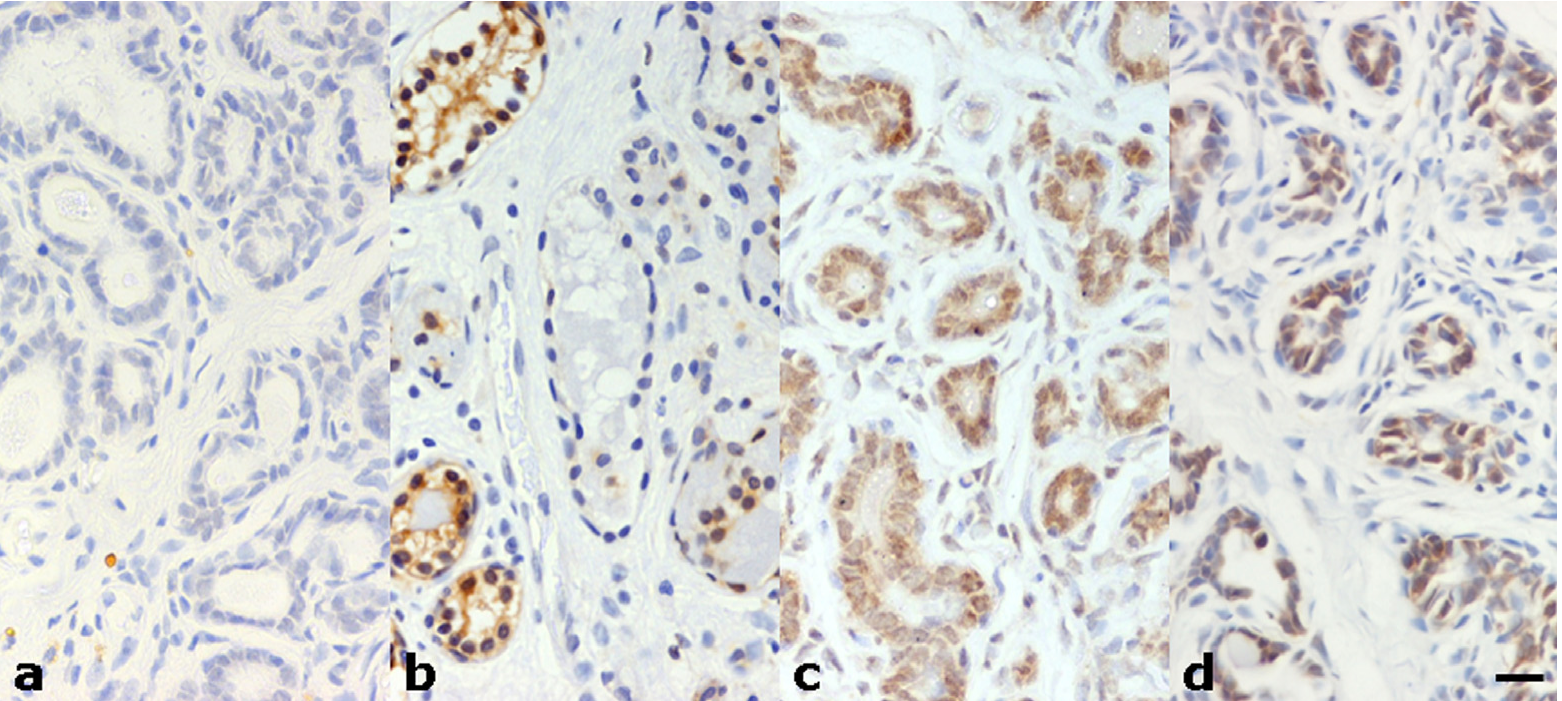
**HSP immunohistochemical expression in normal canine mammary gland tissue**. (a) Hsp27 expression absent; (b) focal Hsp72 expression in both the cytoplasm and nucleus of single epithelial cells and isolated alveolar structures; (c) strong and diffuse Hsp73 immunolabelling; (d) moderately intense and diffuse Hsp90 immunostaining in lobular cells. Scale bar 20 μm.

Hsp27 immunoreactivity was practically absent in normal canine mammary gland (Figure 1a). When a weak immunohistochemical positivity was detected, it was predominantly located in the cytoplasm of the alveolar cells, while both intralobular ductules and extralobular ducts, as well as myoepithelial cells turned out to be negative. A moderate reactivity of endothelial cells was found in blood vessels.

Hsp72 was focally immunodetected in the cytoplasm and nucleus of single epithelial cells or isolated alveolar structures (Figure 1b), while most of the glandular parenchyma was negative.

Hsp73 exhibited a strong and diffuse cytoplasmic immunolabelling in alveolar and ductular cells (Figure 1c) and a less intense and irregular positivity in extralobular ductal structures, while the myoepithelial cells were negative.

Hsp90 showed a moderately intense and diffuse cytoplasmic immunostaining in lobular cells (Figure 1d), whereas extralobular ducts and myoepithelial cells were inconstantly positive. In addition, endothelial cells and smooth muscle tunic of blood vessels often exhibited a moderately intense signal.

### HSP expression in malignant mammary tumours

**Figure 2:**
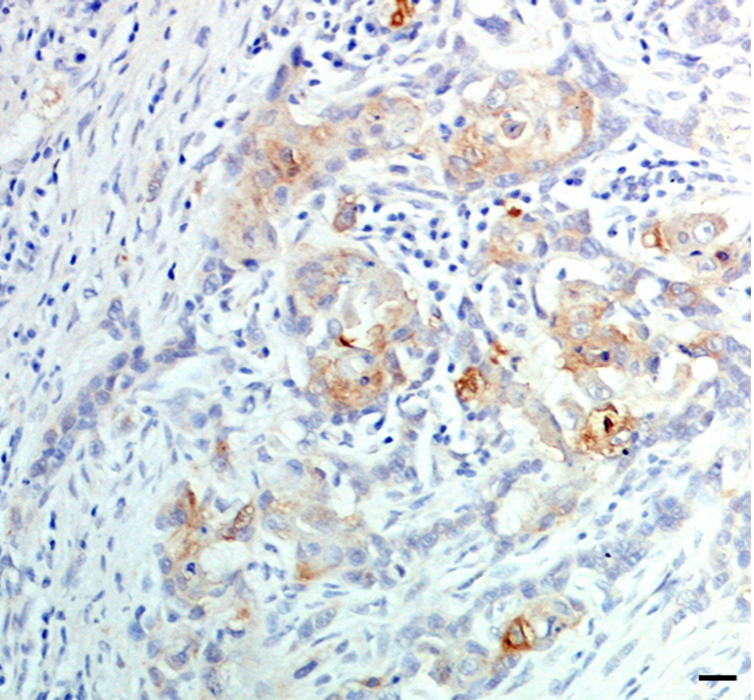
Simple solid carcinoma. Clear Hsp27 positivity in infiltrating tumour cells. Scale bar 30 μm.

**Figure 3:**
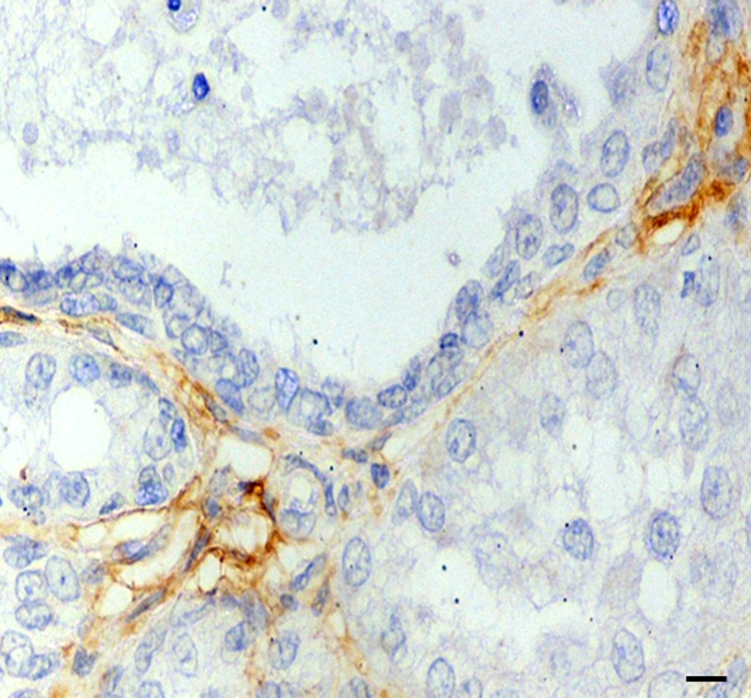
*In situ *carcinoma. Hsp27 immunoreactivity in myoepithelial cells. Scale bar 15 μm.

Hsp27 appeared to be absent or weakly expressed in most tumour cases with no differences among groups; however, in marginal areas of invasive stages, markedly positive single cells or clusters of cells invading the surrounding stroma were frequently observed (Figure 2). A strong Hsp27 immunolabelling of cells with squamous metaplasia was also found. In *in situ *carcinomas, myoepithelial cells showed an intense cytoplasmic signal (Figure 3), which gradually decreased in the infiltrative stages.

**Figure 4:**
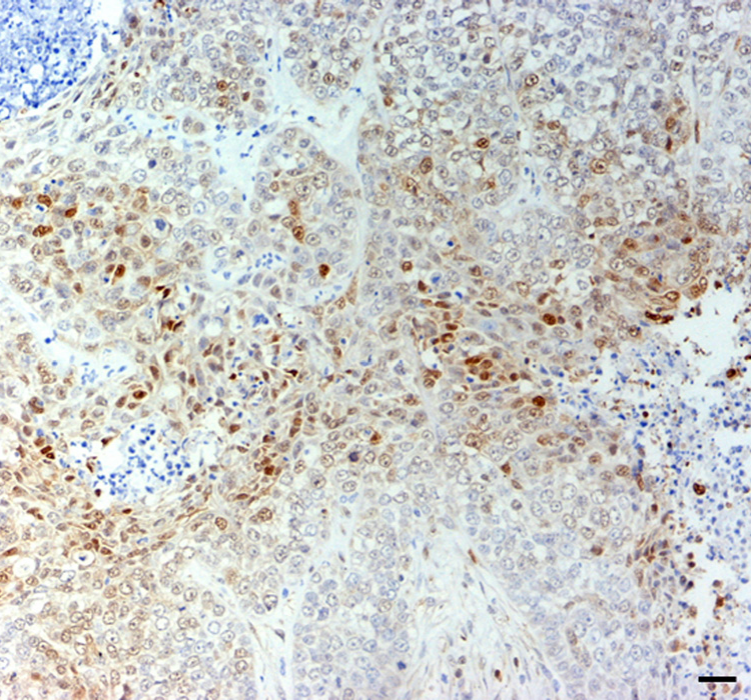
Simple solid carcinoma. Cytoplasmic and nuclear Hsp72 expression of neoplastic cells in an inflammatory area. Scale bar 30 μm.

Hsp72 immunolabelling differed among the various tumours and groups examined, ranging from weak to strong. The highest signal was observed in tumour areas displaying infiltrative growth or surrounding inflammation or necrosis, in which both cytoplasmic and nuclear localization was detected (Figure 4).

**Figure 5:**
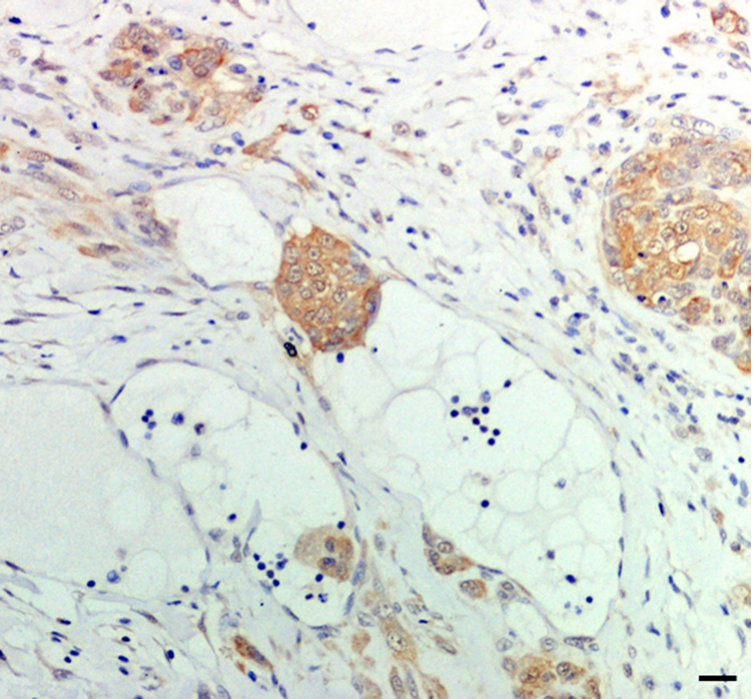
Simple solid carcinoma. Intense Hsp73 immunolabelling in lymphatic neoplastic emboli. Scale bar 30 μm.

Hsp73 was characterized by a gradually reduced intensity of immunostaining in *in situ *and stage I carcinomas, when compared to that of normal mammary tissue. However, in stage II, a highly intense immunoreactivity, mainly located in areas showing infiltrative growth or inflammation, was detected. Cellular elements undergoing mitosis, as well as neoplastic lymphatic emboli also exhibited a strong cytoplasmic positivity (Figure 5).

**Figure 6:**
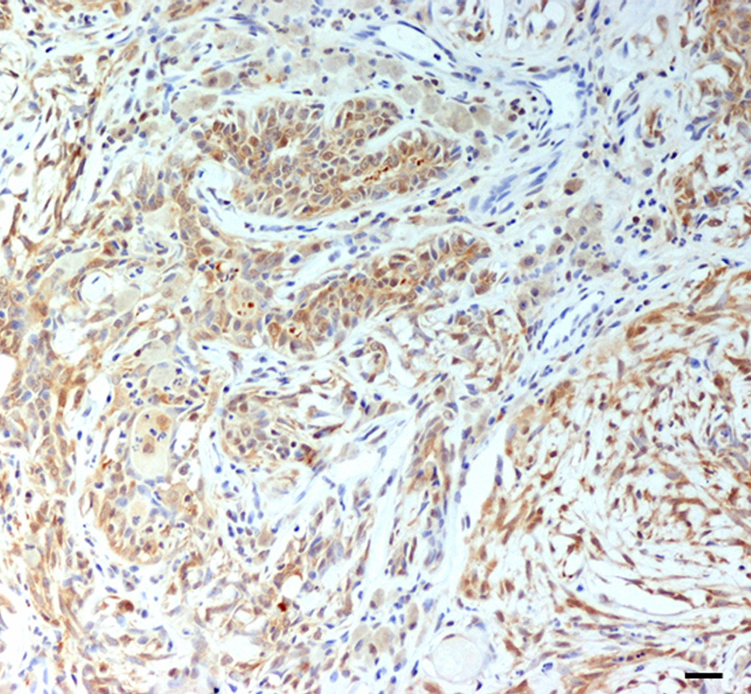
Complex carcinoma. Intense and diffuse Hsp90 immunostaining in both neoplastic elements and proliferating myoepithelial cells. Scale bar 30 μm.

**Figure 7:**
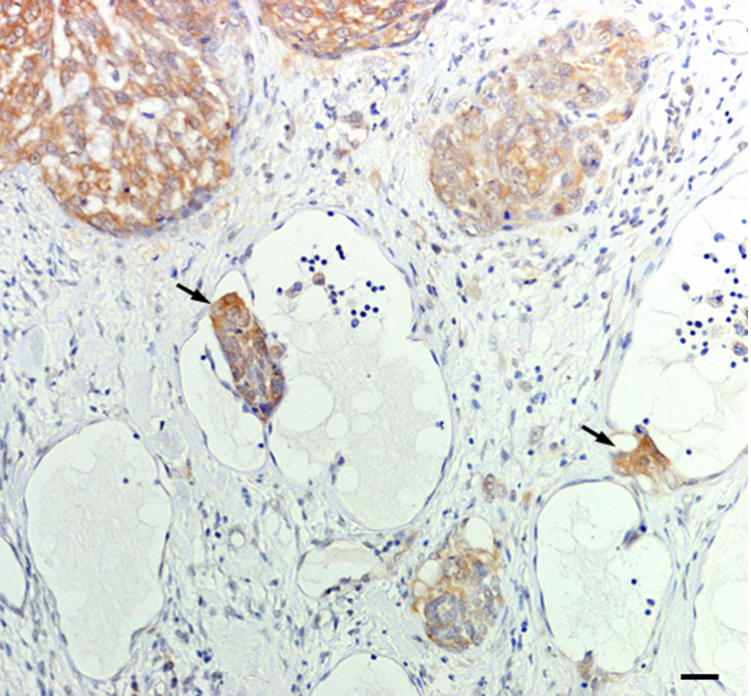
Simple solid carcinoma. Strong Hsp90 positivity in lymphatic neoplastic emboli (arrows). Scale bar 30 μm.

Hsp90 exhibited a strong and diffuse positivity in all tumours examined; normal and proliferating myoepithelial cells, observed in complex carcinoma, also showed an intense immunolabelling (Figure 6). A moderate signal in cells undergoing mitosis and an intense reactivity in lymphatic emboli (Figure 7) were also observed.

The immunoreactivity of the HSPs under study did not show any correlation with a particular histological type of neoplasm in any of the tumour groups examined.

### Comparison of HSP expression in normal mammary gland and malignant mammary tumours

**Figure 8:**
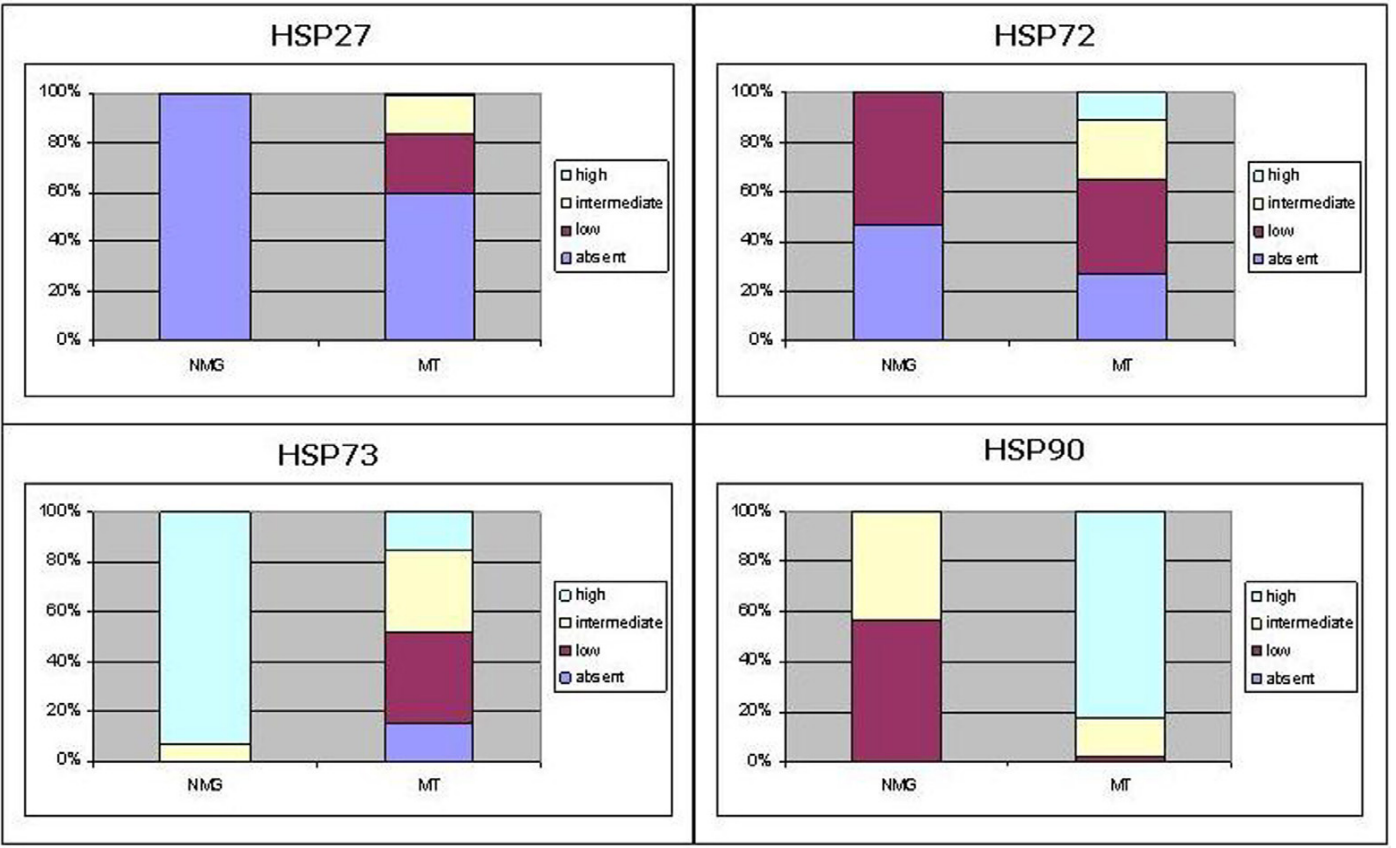
**Comparison of HSP expression in normal mammary glands (NMG) and malignant tumours (MT)**. The figure shows the percentage of expression of each Hsp (absent, low, intermediate and high) in NMG and MT. In MT, a significant increase of Hsp27 (P < 0.01), Hsp72 (P < 0.05) and Hsp90 (P < 0.01) expression, as well as a significant reduction of Hsp73 (P < 0.01) immunoreactivity was found with respect to NMG (Chi square test).

**Figure 9:**
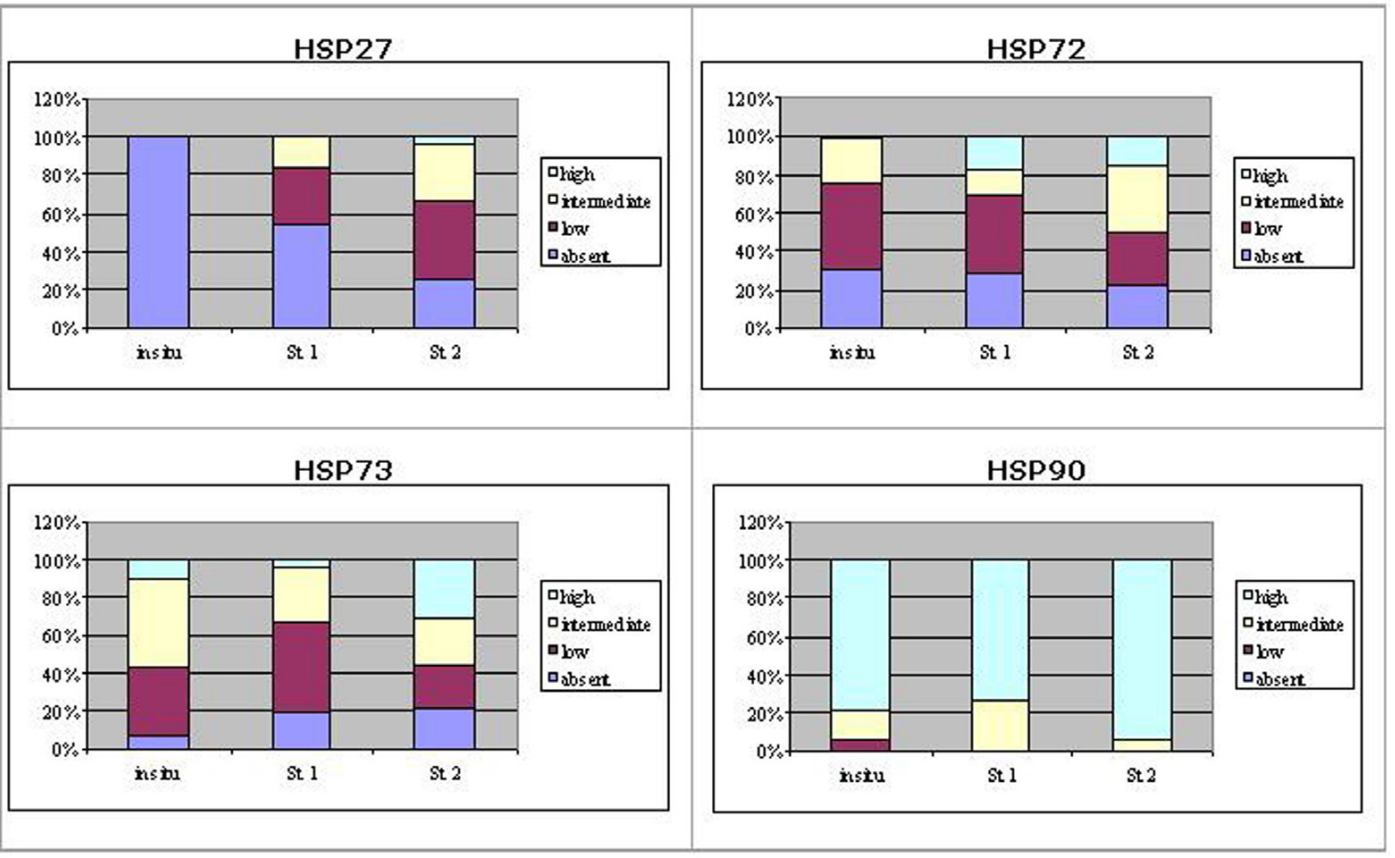
**Comparison of HSP expression among tumour groups**. The figure shows the percentage of expression of each Hsp (absent, low, intermediate and high) in *in situ *and infiltrating malignant tumours, the latter grouped as cases with local stromal invasion (St1) or with intravascular invasion (St2). As far as Hsp27 and Hsp72 expression is concerned, the intermediate and high immunostaining was found to be significantly associated with invasive stages (P < 0.01) (Chi square test). Hsp73 expression did not appear to be correlated to invasion, whereas Hsp90 showed a predominantly high expression in all groups.

In malignant tumours, a significant increase of Hsp27 (P < 0.01), Hsp72 (P < 0.05) and Hsp90 (P < 0.01) expression, as well as a significant fall in Hsp73 (P < 0.01) immunoreactivity was noted compared to normal mammary gland (Figure 8). However when groups were compared, Hsp27 and Hsp72 expression in *in situ *carcinomas appeared to be similar to normal mammary gland, while intermediate and high immunostaining was significantly associated with invasive stages (P < 0.01). Changes in Hsp73 expression did not appear to be correlated to invasion, whereas Hsp90 showed a predominantly high expression in all groups (Figure 9).

### Comparison between HSP expression and OS

**Figure 10:**
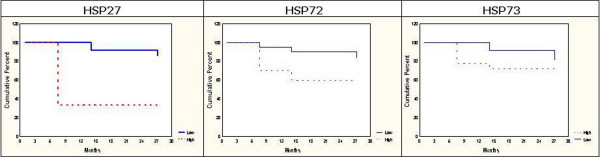
**Survival curves and results of survival analysis**. Influence on survival was established using the Log-Rank Test and cases were grouped according to expression: low (absent + low semiquantitative evaluation) or high (intermediate + high semiquantitative evaluation). Hsp27 appeared to be significantly associated to a shorter post-surgical OS (P = 0.00087), while Hsp72 (P = 0.05291) and Hsp73 (P = 0.1693) did not show a significant correlation to OS.

Survival analysis revealed that Hsp27 was significantly associated to poor prognosis (P = 0.00087), as its high immunodetection appeared to be related to a shorter post-surgical OS. Hsp72 (P = 0.05291) and Hsp73 (P = 0.1693) did not show a significant correlation with OS (Figure 10). The Log-Rank test could not be used for Hsp90 because of its high expression in all cases.

## Discussion

This study shows that, immunohistochemically, several HSPs appear to be expressed in normal canine mammary gland as well as in malignant mammary tumours. In normal tissue, Hsp90 exhibited a moderately intense immunoreactivity, which increased significantly in all the neoplasms examined, independently of invasion or tumour histological type. In human breast cancer, abundant Hsp90α expression has also been observed, which appeared to be closely associated with cell proliferation [31]. In this respect, Hsp90 is known to be involved in the regulation of the cell cycle [32], controlling the activity of several signalling proteins, such as Raf-1 [33], Wee-1 [34] and Akt [35] kinases. In breast cancer cells, Hsp90 is also essential for the stability and function of steroid hormone receptors [36], as well as the membrane receptor tyrosine kinase ErbB2 [37], whose enhanced expression correlates with malignancy of breast cancer progression [25] and which might also play an important role in carcinogenesis of canine mammary gland [38-41]. Hsp90α expression has also been reported to indicate a poor prognosis in human breast cancer [12], pancreatic carcinoma [42], and acute leukaemia [43]. However, we were unable to find any prognostic significance for this Hsp probably as a result of its high immunoreactivity in nearly all of the cases and in all of the groups examined. Notwithstanding this, these results suggest that, given its high expression levels in neoplastic tissues, Hsp90 could hold a fundamental role in the multiple processes leading to carcinogenesis and tumour progression in the canine mammary gland. Several *in vitro *[44-47] and *in vivo *studies [45-48], based upon the use of Hsp90-binding agents, such as geldanamycin derivatives, have indicated this protein as an alternative molecular target for human breast cancer therapy. In canine mammary tumours, the immunohistochemical detection of estrogen receptor α has been indicated as a possible parameter for selecting appropriate future treatment protocols [28]. To our knowledge, the immunohistochemical expression of HSPs in these neoplasms has not been reported in veterinary literature. However, a recent study has demonstrated a similar pattern of changes in HSPs and apoptosis-associated proteins in both human and canine mammary tumours, which lends weight to the use of the canine model to clarify the molecular mechanisms of mammary carcinogenesis [49]. In particular, Hsp70 and Hsp90 expression, investigated using Western Blotting, appeared to be significantly higher in both human and canine neoplasms [49], confirming our immunohistochemical results. The intense Hsp90 immunoreactivity detected in our cases suggests that this protein could represent a novel molecular target for adjuvant cancer treatment of canine mammary tumours, and this animal model could be studied for further testing new breast cancer therapy. In addition, since adjuvant treatments are mainly aimed at controlling micrometastases, the strong Hsp90, as well as Hsp73, immunolabelling detected in neoplastic emboli appears to be remarkable, as it indicates that these HSPs are necessary to cells with metastatic potential and that the inhibition of their functions could affect the survival of such cells, which do not always show the same pattern of expression respect to the primary tumour [50]. The need for valid in vivo models for further testing Hsp90-targeted cancer therapy, currently under clinical trial with promising early results [22,51-53], also appears to be essential, as a recent study has highlighted a potential contraindication to this therapy, since the Hsp90 inhibitor, 17-allylamino-17-demothoxygeldanamicin (17-AAG), appears to enhance bone metastasis of a human breast cancer cell line following intracardiac inoculation in the nude mouse [54].

In our study, both Hsp90 and Hsp73 exhibited a clear-cut expression in mitotic cells: Hsp90 seems to regulate the metaphase-anaphase transition [55], by controlling the stability of the centrosomal Polo kinase [56], whilst Hsp73 appears to be localized on the fibres of spindles and asters during metaphase [57].

Hsp73 also appeared to be constitutively expressed with strong immunolabelling in normal canine mammary glands. This finding could be related to its association with glandular intermediate filaments, such as keratin polypeptides 8 and 18 [58] and the gradually reducing Hsp73 immunoreactivity, detected in *in situ *and stage I carcinomas, could be related to a partial loss of differentiation in neoplastic cells, which could determine a reduced expression of typical normal glandular tissue proteins.

On the other hand, the high positivity of HSP70 family members, frequently detected in areas of intense proliferative activity and/or stromal invasion, could be correlated to the roles exerted by these chaperones in cell cycle control [32] or environmental stress such as hypoxia [59], suffered by these cells, particularly in the more aggressive tumour areas. Furthermore, in some cases, the presence of an inflammatory reaction surrounding groups of strongly positive cells could explain their intense immunoreactivity, given that the mediators of inflammation are able to induce HSPs synthesis and increased HSP expression protects cells from the cytotoxic effects of these substances [60,61].

Hsp27 appeared to be absent or weakly expressed in normal canine mammary gland, similarly to humans [2,62]. However, we only observed weak and inconstant expression of this Hsp in most tumour cases contrary to human breast cancer in which high levels of Hsp27 have frequently been found [8,10,13,63,64]. Nevertheless, in stage I and II, infiltrating neoplastic cells, particularly those peripheral ones often showed an intense immunoreactivity for this Hsp. In fact a strict correlation between Hsp27 over-expression and invasiveness of human breast cancer cells has been observed in *in vitro *and *in vivo *studies demonstrating that this protein may influence the invasive and metastatic potential of these cells [65], probably by controlling their migration on laminin-5 [66]. Furthermore, a synthetic inhibitor of protein kinase C-dependent phosphorylation of Hsp27 has recently been demonstrated to block tumour cell migration and invasion [67]. Hsp27 is known to interact with several cytoskeletal proteins, thus playing an important role in assembly, remodelling, as well as protection under stress of the actin cytoskeleton [68-70]. In our cases, these functions could explain Hsp27 immunoreactivity not only in infiltrative tumour elements, but also in myoepithelial cells, which also showed Hsp90 positivity. Similar results have been obtained in the study of human adult salivary glands, in which the involvement of these HSPs in the control of the organization of the cytoskeleton was hypothesized [71]. However, in this study an explanation for the lack of Hsp27 immunodetection in mioepithelial cells of normal tissue, as well as for the fall in expression intensity from *in situ *to stage II carcinomas, was not given. In addition, the opposite Hsp27 immunoreactivity detected in myoepithelial cells of normal mammary gland and in those ones of *in situ *tumours is unexpected, since *in situ *malignant proliferations are typically characterized by an intact myoepithelial cell layer, as in normal tissue [72].

We also observed a strong Hsp27 immunoreactivity in keratinising cells of tumour areas showing squamous metaplasia, thus confirming our recent study, which indicated that Hsp27 should be regarded as a differentiation marker for keratinocytes, both in normal and in neoplastic canine skin [73]. In fact this Hsp exerts prominent roles in the process of keratinization [74,75].

Finally, only Hsp27 seems to have a prognostic significance for survival in these canine neoplasms; its high immunodetection in invasive tumour stages appears to be indicative of a poorer clinical outcome, represented in our cases by a shorter post-surgical OS. In human medicine, several studies have looked at the prognostic significance of Hsp27 in breast cancer [10,11,13,15,17], however producing conflicting results.

## Conclusion

Given their increased immunoreactivity in neoplastic tissues, we believe that Hsp27, Hsp72 and Hsp90 may play a role in carcinogenesis of the canine mammary gland. The immunodetection of Hsp90 in tumour cells during metastatic spread confirms the importance of this protein as a molecular target for adjuvant cancer therapy and highlights the possible usefulness of the canine model for studying human breast cancer. The absent to weak Hsp27 expression both in normal mammary tissue and in *in situ *carcinomas, on the other hand, indicates that this protein is not directly implicated in the physiology or neoplastic transformation of the canine mammary gland. However the high Hsp27 immunohistochemical expression showed by infiltrating tumour cells suggests that this Hsp is involved in tumour invasiveness and is indicative of a poor prognosis. To the best of our knowledge the present study, although preliminary, is the first report of HSP immunohistochemical expression in normal and neoplastic canine mammary glands.

## Competing interests

The author(s) declare that they have no competing interests.

## Authors' contributions

MR participated in the design of the study, carried out the immunohistochemistry and drafted the manuscript. AM carried out the immunohistochemistry. GS performed the statistical analysis. LDS conceived the study, and participated in its design and coordination and helped to draft the manuscript. All authors read and approved the final manuscript.

## Pre-publication history

The pre-publication history for this paper can be accessed here:


